# Person‐centred, integrated non‐communicable disease and HIV decentralized drug distribution in Eswatini and South Africa: outcomes and challenges

**DOI:** 10.1002/jia2.26113

**Published:** 2023-07-06

**Authors:** Deborah Goldstein, Nathan Ford, Nicholas Kisyeri, Maggie Munsamy, Lirica Nishimoto, Kufor Osi, Herve Kambale, Thomas Minior, Moses Bateganya

**Affiliations:** ^1^ Office of HIV/AIDS USAID Washington DC USA; ^2^ Global HIV Hepatitis and Sexually Transmitted Infections Programmes World Health Organization Geneva Switzerland; ^3^ Eswatini National AIDS Program Mbabane Eswatini; ^4^ ICAP Columbia University Mbabane Eswatini; ^5^ National Department of Health Pretoria South Africa; ^6^ FHI 360 Durham North Carolina USA; ^7^ Resolve to Save Lives Abuja Nigeria; ^8^ ICAP Columbia University Mailman School of Public Health New York New York USA

**Keywords:** non‐communicable disease, integration, person‐centred care, decentralized drug distribution, HIV, hypertension, diabetes

## Abstract

**Introduction:**

Non‐communicable diseases (NCDs) are highly prevalent in people living with HIV above 50 years of age and account for increasing mortality. There is little published evidence supporting person‐centred, integrated models of HIV care, hypertension and diabetes treatment in southern Africa, and no data demonstrating mortality reduction. Where clinical visits for NCDs and HIV cannot be combined, integrated medication delivery presents an opportunity to streamline care and reduce patient costs. We present experiences of integrated HIV and NCD medication delivery in Eswatini and South Africa, focusing on programme successes and implementation challenges. Programmatic data from Eswatini's Community Health Commodities Distribution (CHCD) from April 2020 to December 2021 and South Africa's Central Chronic Medicines Dispensing and Distribution (CCMDD) from January 2016 to December 2021 were provided by programme managers and are summarized here.

**Discussion:**

Launched in 2020, Eswatini's CHCD provides over 28,000 people with and without HIV with integrated services, including HIV testing, CD4 cell count testing, antiretroviral therapy refills, viral load monitoring and pre‐exposure prophylaxis alongside NCD services, including blood pressure and glucose monitoring and hypertension and diabetes medication refills.  Communities designate neighbourhood care points and central gathering places for person‐centred medication dispensing.  This programme reported fewer missed medication refill appointments among clients in community settings compared to facility‐based settings. South Africa's CCMDD utilizes decentralized drug distribution to provide medications for over 2.9 million people, including those living with HIV, hypertension and diabetes.  CCMDD incorporates community‐based pickup points, facility “fast lanes” and adherence clubs with public sector health facilities and private sector medication collection units.  There are no out‐of‐pocket payments for medications or testing commodities.  Wait‐times for medication refills are lower at CCMDD sites than facility‐based sites.  Innovations to reduce stigma include uniformly labelled medication packages for NCD and HIV medications.

**Conclusions:**

Eswatini and South Africa demonstrate person‐centred models for HIV and NCD integration through decentralized drug distribution. This approach adapts medication delivery to serve individual needs and decongest centralized health facilities while efficiently delivering NCD care.  To bolster programme uptake, additional reporting of integrated decentralized drug distribution models should include HIV and NCD outcomes and mortality trends.

## 1 INTRODUCTION

In low‐ and middle‐income countries, 15% of people living with HIV are aged 50 years and older [1]. Non‐communicable diseases (NCDs) have increased in prevalence among older people living with HIV [2] with a significant impact on health and increasing contributions to mortality [3]. NCD prevalence among people living with HIV in low‐ and middle‐income countries includes 21% hypertension, 22% hypercholesterolemia and 1.3%–26% diabetes [4]. A multicentre cohort study of U.S. President's Emergency Plan for AIDS Relief (PEPFAR)‐supported clinical sites in Kenya, Uganda, Tanzania and Nigeria from 2011 to 2021 found up to 30.5% had elevated blood pressure and up to 15.8% had dysglycaemia; additionally, the prevalence of NCDs increased among people older than age 50 years living with and without HIV [5].

Despite increasing NCD prevalence, there is a lack of high‐quality published evidence to support person‐centred NCD/HIV integration models in southern Africa [6, 7, 8]. A World Health Organization guideline process in 2021 identified implementation studies of the integration of hypertension and diabetes care with differentiated models of HIV service delivery as a specific research gap [9]. Integration may increase access to hypertension and diabetes care among people living with HIV with limited access to primary preventive services [10, 11] and may potentially improve health outcomes and reduce mortality [3, 12].

Person‐centred programming places individuals’ values and preferences at the centre of all aspects of programme design and implementation. To promote awareness of successful large‐scale NCD/HIV integration projects, we present experiences with person‐centred models for HIV and NCD integration through decentralized drug distribution from Eswatini and South Africa, focusing on programmatic successes and implementation challenges. Programmatic data from Community Health Commodities Distribution (CHCD) in Eswatini and Central Chronic Medicines Dispensing and Distribution (CCMDD) in South Africa were provided by programme managers and are summarized for Eswatini, from April 2020 to December 2021, and for South Africa, from January 2016 to December 2021.

## 2 DISCUSSION

### 2.1 Eswatini: Community Health Commodities Distribution

#### 2.1.1 Programme design

In April 2020, the Eswatini Ministry of Health responded to the COVID‐19 pandemic by creating CHCD, building upon differentiated service delivery (DSD) models within their national Community Commodity Distribution framework, to ensure ongoing access to medications while minimizing client risk [13]. With 11 implementing partners supported by the Government of Eswatini, PEPFAR, the Global Fund and Médecins Sans Frontières, CHCD provides medication distribution, community engagement and supply chain management for community‐based DSD models, including community antiretroviral therapy (ART) groups [14].

CHCD initially used HIV medication distribution as the entry point due to the existing foundation of the HIV programme. As a response to client perceptions of HIV stigma, however, NCDs, family planning and other services were later added at some facilities, allowing people living with HIV to access these medications, and allowing those without HIV to also participate in CHCD.

CHCD services currently include HIV testing, ART, CD4 cell count, viral load monitoring, adherence support services and pre‐exposure prophylaxis alongside NCD services, including blood pressure and weight monitoring, glucose testing, hypertension and diabetes medication refills, sexually transmitted infection screening, family planning and treatment of minor ailments. Participants living with HIV must be 18 years or older, on ART for at least 12 months with two undetectable viral loads, on a first‐line antiretroviral (ARV) regimen, not pregnant, and agree and consent to participate. There are no out‐of‐pocket costs for CHCD participation and transportation subsidies are not offered.

Eligible clients receive decentralized services either at small, convenient public health facilities or at decentralized pickup points (PUPs). At both facility and community levels, Expert Clients and community leaders identify secure locations of community distribution points within catchment areas; clients then select the most convenient site for ART pickup. Neighbourhood PUPs are at central gathering places, including under a tree, schools, churches, bus stops, community halls, football pitches, shops and local government offices.

Expert Clients at the community level also coordinate with client support groups to recruit clients established on ART. Client information is first recorded in a paper CHCD register, then transferred into electronic client management software. Eswatini's Ministry of Health adapted the national electronic Client Management Information System to enable tracking of data relevant to DSD and conducted annual assessments to evaluate coverage, quality and impact of DSD models [15].

Nurses and Expert Clients at CHCD facilities pre‐pack ART and NCD medications and carry them to community distribution points. Multi‐month dispensing of 3–6 months of ART is aligned with up to 3 months for NCD medications, depending on stock availability. After their vital signs are taken, clients are screened for COVID‐19, tuberculosis and other illnesses. Viral load specimens are obtained where possible, or ART clients are referred to a health facility.

The proportion of health facilities implementing DSD models grew from 13% in 2016 to 96% in 2020, and the diversity of these models increased over time, currently with five facility‐based and two other community‐based models in addition to CHCD; there are also tailored models for adults and adolescents living with HIV, individuals with comorbidities and advanced HIV disease, men, pregnant and breastfeeding women, high viraemic, and key and vulnerable populations. The proportion of people on ART enrolled in less‐intensive DSD models increased from 7.9% in 2017 to 80.4% in 2020. Viral load suppression increased from 90% in males and 91% in females in 2017 to 96% and 97%, respectively in 2020 [15].

The Ministry of Health medication‐dispensing locker pilot has started in high‐volume sites in two regions and will evaluate viral suppression among HIV/NCD clients and the extent of hypertension and diabetes control. These findings will inform future directions, including potentially involving private pharmacies.

#### 2.1.2 Programme outcomes

Eighty‐three PEPFAR‐supported facilities and 721 functional community distribution points implemented CHCD during the study period. The 26,776 clients enrolled represent people living with and without HIV; 63% female and 4% below 15 years of age. Accounting for additional clients who spontaneously present to care, a total of 28,851 clients received medicines through CHCD in Eswatini during the study period. Less than 10% of all clients receiving ART from facilities that conduct CHCD collected medications from PUPs. Only 1% of CHCD clients missed appointments in this programme compared to 7% who missed appointments for facility‐based refills.

#### 2.1.3 Programme challenges

The low percentage of clients collecting ART from PUPs is thought due to perceived HIV‐related stigma as CHCD was initially designated only for people living with HIV. While client preference for facility‐based refills has decreased with the standardized integration of glucose testing and hypertension medication refills across CHCD sites, some people living with HIV who require more comprehensive medical management continue to prefer facility‐based care.

Due to supply chain difficulties resulting in low NCD medication supply, clients sometimes purchase from private pharmacies or receive only 1‐month supplies from CHCD.

The lack of funding for client transportation is another challenge. Financial sustainability is an ongoing challenge as much of its funding is from implementing partners.

### 2.2 South Africa: Central Chronic Medicines Dispensing and Distribution

#### 2.2.1 Programme design

Launched in 2014, South Africa's National Department of Health's Central Chronic Medicine Dispensing and Distribution (CCMDD) flagship programme for national health insurance provides people with well‐controlled NCDs and people living with HIV with virologic suppression with medications to control chronic diseases, including HIV via community‐based PUPs, facility “fast lanes” and adherence clubs. Now expanded nationally, the programme has more than 4.9 million clients registered [16]. CCMDD is fully funded through the South African government, the Global Fund and PEPFAR, ensuring no out‐of‐pocket payments for medications or testing commodities. Transportation subsidies are not offered.

CCMDD governance incorporates the Ministerial National Essential Medicines List Committee, the Pharmaceutical Therapeutics Committees, a National CCMDD Task Team, provincial and district task teams, and facilities committees. CCMDD uses public‐sector health facilities, 6‐month medication prescriptions and private‐sector partnerships to promote programme sustainability. Through contracted private sector central medication dispensing and private couriers, it reaches 2855 PUPs at independent and community pharmacies, doctors’ rooms, smart lockers and participating retailers.

CCMDD eligibility criteria include people ages 18 years and older, on medical treatment for at least 6 months, clinician confirmation of client eligibility and client's voluntary desire to participate; tuberculosis or other medical conditions requiring regular clinical consultation are exclusionary [17].

Pre‐dispensed medications are provided through differentiated models, facility‐ or community‐based adherence clubs and external PUPs, including private pharmacies, lockers and community points [18]. Person‐centred innovations include clients selecting their own language for written medication instructions; clients deciding where they want to collect their medicine parcel; clients choosing the exact date of medication pickup (within a 7‐day window); and client feedback informing the design of medication packaging and labelling.

Community representatives identify potential PUPs, provide input to programmatic standard operating procedures, ensure that client concerns are responded to and ensure that medication shortages are promptly reported.

A recent multisite study of 1642 CCMDD participants living with HIV who were virally suppressed at the time of enrolment evaluated the impact of PUP type on maintaining 12‐month virologic suppression. The choice of PUP, whether community‐based ART PUPs (i.e. private pharmacies, schools and churches) or facility‐based ART PUP (separate fast‐track lane), was not significantly associated with virologic suppression, with 86% of participants with viral load data available maintaining viral suppression [19].

CCMDD aimed to reduce perceived stigma for people living with HIV in participating private pharmacies by integrating NCD/ART medication pickup, using uniformly labelled medication packages, and ensuring visual and audio privacy in dispensing areas [20].

#### 2.2.2 Programme outcomes

By October 2021, more than 3.2 million people were registered on the CCMDD programme with 2,645,945 (83%) recipients receiving ART and other chronic disease medications, and 556,625 (17%) receiving only chronic disease medications [18]. The most commonly prescribed NCD medications for people living with HIV are for hypertension (35.4%), diabetes (12.0%) and angina (8.7%); less commonly prescribed medications are for asthma/chronic obstructive pulmonary disease, chronic kidney disease and mental health disorders (Table 1).

**Table 1:** Most common comorbidities among people living with HIV on ART in South Africa's Chronic Medicines Dispensing and Distribution: January, 2016–December, 2021 (total exceeds 100% as some people obtain medications for more than one NCD)

Condition	Number of people living with HIV who also received medications for other conditions (% of total 2,645,945 people living with HIV enrolled in CCMDD during this period)
Hypertension	936,564 (35.4%)
Diabetes	316,707 (12.0%)
Angina	229,098 (8.7%)
Isoniazid preventive therapy	116,028 (4.4%)
Osteoarthritis	87,500 (3.3%)
Ischaemic heart disease	82,170 (3.1%)
Pain	58,493 (2.2%)
Congestive cardiac failure	42,101 (1.6%)
Asthma	32,690 (1.2%)
Hyperlipidaemia	31,259 (1.2%)
Epilepsy	27,950 (1.1%)
Depressive disorder	12,697 (0.5%)
Family planning	9999 (0.4%)

Abbreviations: ART: antiretroviral therapy; NCD: non‐communicable disease; CCMDD, Central Chronic Medicine Dispensing and Distribution.

Less than 1% of prescriptions for people living with HIV are for family planning and depressive disorder; plans to scale up prescribing for these components are underway. Isoniazid preventive therapy accounts for only 4.4% of prescriptions for people living with HIV because most of these clients have already completed this therapy by the time of CCMDD enrolment. Future plans include incorporating insulin and tuberculosis screening and treatment into CCMDD.

By October 2021, among those receiving ART and other chronic disease medications, 25% utilized facility PUPs, 19% participated in adherence clubs and 56% used external PUPs. Of note, many clients originally assigned to adherence clubs in the pre‐COVID era collected medications through facility PUPs due to limitations of COVID‐19 lockdown regulations discouraging social gatherings during this timeframe [18]. Community‐based PUPs were prioritized in response to COVID‐19 precautions, with the proportion of clients using community‐based PUPs increasing from 36% to 56% from January 2020 to May 2021 [17].

Seventy‐seven percent of CCMDD clients report less than 5‐minute waiting times, compared to average wait times of 4–6 hours at public facilities.

#### 2.2.3 Programme challenges

CCMDD has been transitioning to web‐based electronic prescribing because hand‐written prescriptions were found to be more frequently rejected for incorrect medication dosage, incomplete instructions, illegibility, lack of clinician signature or because they were written for a medication that was not on the CCMDD formulary. Web‐based prescriptions have significantly decreased the amount of rejected prescriptions. To date, the electronic system has only been rolled out to certain Districts, but funds have been secured for 2023–2024 to roll out the electronic system to the remaining 1026 facilities.

South Africa's energy crisis causing disruptions in electricity availability negatively impacted the repacking of electrified smart lockers, and additional batteries were added to these units. The lack of internet connectivity in some facilities is another country challenge which impacts the use of web‐based prescribing.

Client feedback collected through monitoring and evaluation revealed people living with HIV experienced stigma from private sector site employees, including negative attitudes from staff when handing out medication parcels, and CCMDD clients being made to wait while private sector clients were serviced first. Specific PUPs were identified and client perceptions of stigma were discussed during routine quarterly meetings with each PUP team. Trainings were implemented for the affected PUP teams and sites were monitored for improvement. If client feedback did not improve, the PUPs were considered for closure. Subsequent monitoring and evaluation have shown marked improvements in perceptions of service.

Additional challenges at private sector sites included the lack of sufficient storage space, particularly at popular PUPs with more than 2000 clients. When PUPs reach capacity, they are temporarily unavailable in the electronic system until storage issues stabilize.

## 3 CONCLUSIONS

Decentralized drug distribution, a client‐centred approach that adapts medication delivery to better serve individual needs and decongest centralized health facilities, permits the efficient integration and delivery of NCD care and has the potential to reduce healthcare costs borne by clients with multiple comorbidities. Eswatini and South Africa demonstrate scalable programmes for HIV and NCD integration through decentralized drug distribution programming. Public−private partnerships among government agencies, health ministries, non‐governmental agencies, federal programmes and international donors were crucial to the success of both programmes, as was the absence of out‐of‐pocket costs to clients in both programmes.

Because of the different time periods of data available, direct inter‐programme comparisons are not possible. However, one common theme is both programmes’ use of HIV and NCD medication co‐dispensing to reduce HIV‐related stigma around use of community PUPs. In Eswatini's programme, initially designated for people living with HIV without comorbidities, clients were at first discouraged from utilizing community‐based PUPs due to perceived stigma, but NCD care was included with the hope of decreasing stigma and improving uptake. In South Africa, despite CCMDD's provision of both ART and NCD medications for people living with HIV since programme inception, clients still reported HIV stigma from providers at private sector sites; this was later resolved with staff training. Integration of ART and NCD medications alone is not sufficient to address HIV‐related stigma, and investments in trainings and close monitoring and supervision are necessary. However, the integration of services may help alleviate the challenges.

Another factor in both programmes’ success was the use of community leaders and Expert Clients in the community throughout programme design and implementation—to select PUP sites; to enrol clients; and to ensure client complaints were adequately addressed—which allowed for person‐centred service delivery models that are continuously adapted and modified to respond to client needs, comfort and convenience.

These data confirm the high prevalence of hypertension and diabetes, among other NCDs, among people living with HIV in South Africa's CCMDD. However, this analysis is limited by a lack of data from Eswatini's CHCD on clients’ HIV status and by the lack of NCD prevalence data among people living with HIV. Additional analysis is needed to better understand factors contributing to Eswatini's low rate of PUP utilization for ART clients; to evaluate whether programme expansion to include people living with HIV with well‐controlled comorbidities has increased PUP utilization for ART; and to identify other successful strategies to increase PUP use. Future studies should evaluate the impact of sex on programme participation and HIV outcomes data; provide HIV outcomes data; and detail the programmatic impact on HIV retention in care.

Further evaluation to help inform other countries’ plans for integrated HIV/NCD drug distribution programmes includes analysis of factors correlated with improved retention in care; patterns of service uptake or site preferences among people living with HIV compared to people with NCDs; analysis of HIV clinical outcomes by mode of decentralized drug distribution (DDD) programme utilized and by demographic factors, including gender and age, including risk factors for loss of viral suppression; and client preference data on stigma, provider trust and factors influencing the choice of facility type.

PEPFAR's infrastructure is successfully utilized to support NCD and HIV integration through decentralized drug distribution, including the establishment of data systems, supply chain, policies and regulations to support dispensing outside of facilities; joint training systems and materials; demand creation; and engaging stakeholders. To bolster programme uptake in southern Africa, additional reporting of integrated NCD and HIV decentralized drug distribution models is needed on client‐level clinical outcomes, including blood pressure and glycaemic control, HIV, including 95‐95‐95 benchmarks, and longer‐term mortality trends through modelling studies.

## COMPETING INTERESTS

No competing interests to report.

## AUTHORS’ CONTRIBUTIONS

DG: conceptualization; writing—original draft; writing—review & editing. NF: writing—review & editing. NK: investigation; writing—review & editing. MM: investigation; writing—review & editing. LN: writing—review & editing, project administration. KO: writing—review & editing. HK: writing—review & editing. TM: conceptualization, writing—review & editing. MB: Supervision. writing—review & editing. All authors have approved the final version of the manuscript.

## DISCLAIMER

This does not represent the opinion of USAID or the U.S. Government.
